# Prototypes of Newly Conceived Inorganic and Biological Sensors for Health and Environmental Applications

**DOI:** 10.3390/s121217112

**Published:** 2012-12-12

**Authors:** Claudio Nicolini, Manuela Adami, Marco Sartore, Nicola Luigi Bragazzi, Valter Bavastrello, Rosanna Spera, Eugenia Pechkova

**Affiliations:** 1 Laboratories of Biophysics and Nanobiotechnology, Department of Experimental Medicine (DIMES), University of Genoa, Genoa 16132, Italy; E-Mails: nicola@genethics.ethicsoft.it (N.L.B.); vbavastrello@gmail.com (V.B.); rspera@ibf.unige.it (R.S.); epechkova@ibf.unige.it (E.P.); 2 Nanoworld Institute Fondazione EL.B.A. Nicolini, Bergamo 24100, Italy; 3 Elbatech Srl, Marciana, Marciana 57030, Italy; E-Mails: adami@elbatech.com (M.A.); sartore@elbatech.com (M.S.)

**Keywords:** QCM_D, protein-protein interaction, calcium oxide matrices, CO_2_, Langmuir-Blodgett, laccase, amperometry, clomipramine

## Abstract

This paper describes the optimal implementation of three newly conceived sensors for both health and environmental applications, utilizing a wide range of detection methods and complex nanocomposites. The first one is inorganic and based on matrices of calcium oxide, the second is based on protein arrays and a third one is based on Langmuir-Blodgett laccase multi-layers. Special attention was paid to detecting substances significant to the environment (such as carbon dioxide) and medicine (drug administration, cancer diagnosis and prognosis) by means of amperometric, quartz crystal microbalance with frequency (QCM_F) and quartz crystal microbalance with dissipation monitoring (QCM_D) technologies. The resulting three implemented nanosensors are described here along with proofs of principle and their corresponding applications.

## 1. Introduction

This paper describes the utilization of a wide range of complex nanocomposites [1] for optimal implementation of three newly conceived sensors within the framework of FIRB Nanoitalnet. We used inorganic nanocomposites, such as matrices of calcium oxide [2], and biological ones such as Nucleic Acid Programmable Protein Arrays (NAPPA) [3,4] along with Langmuir-Blodgett (LB) multi-layers of proteins of primary interest such as laccase [5,6]. Special attention was paid to the detection of substances significant for both the environment (such as carbon dioxide) and medicine (drugs and cancer) by means of a wide variety of detection methods (amperometric, conductometric, and nanogravimetric).

The resulting three organic and biological constructed nanosensors, here presented in their final versions, were optimized on the outcome of their proof of principles studies and applied for three pilot cases, two for health use and one for the environment. However, all three nanosensors have prominent medical implications, since it is well known that there is a strong link between environment and health and that, according to the WHO (World Health Organization), at least 25% of diseases are due to environmental risk factors.

Sensors for detection of CO_2_[2] can be useful to reveal the increased emissions of gases from fossil fuel combustion, industrial processes, and agriculture associated to deforestation and habitat destruction. This can result in consequent changes in the chemical composition of the atmosphere with direct biological effects and negative influence on the Earth’s climate [2] considering that fossil fuel CO_2_ emissions can remain in the atmosphere for periods of up to tens of thousands of years. The instruments generally employed in these determinations basically consist of infrared (IR) detectors performing a continuous plotting in the site with a good degree of accuracy, but they cannot be used for extensive mapping work because of the large number of expensive devices and specialized people that would have to be involved. Long-term sampling devices such as diffusive sampling techniques are the cheapest and easiest way.

An alternative long-term sampling method for the determination of environmental CO_2_ accumulation takes advantages of the properties of CaO to be carbonated by this gas as shown in reference [2], according to the following equation:

(1) $$\mathrm{CaO}+{\mathrm{CO}}_{2}⇄{\mathrm{CaCO}}_{3}$$

For this purpose, we studied here the variation of mass connected to the carbonation process in order to assess the quantity of gas absorbed by a fixed amount of composite in relation to the concentration of environmental CO_2_.

For what concerns the two health prototypes [3,5], one biosensor of new conception was realized by coupling the traditional quartz crystal microbalance with frequency monitoring (QCM_F) [7] with the quartz crystal microbalance with dissipation monitoring (QCM_D) [8,9], and with an innovative protein cell-free expression system named Nucleic Acid Programmable Protein Arrays (NAPPA) [10,11], that allowed us to immobilize on the quartz surface, as sensing molecule, any kind of protein [3]. In the QCM_D sensor [12] each quartz contained 4×4 NAPPA spots of 300-micron square and functional proteins were synthesized *in situ* directly from printed cDNAs (complementary DNAs) just before the assay [4,12]. Standard nanogravimetry exploited the piezoelectric quartz crystals’ properties to vary the resonance frequency, *f,* when a mass, *Δm,* was adsorbed to or desorbed from their surface according to the Sauerbrey equation:

(2) $$\frac{\Delta f}{f_{0}}=-\frac{\Delta m}{A\rho l}$$

where *f_0_* is the fundamental frequency, *A* is the gold area and *ρ* and *l* are the quartz density and thickness, respectively [7–9]. Quartz resonators used in fluids are more than mere mass or thickness sensors; sensor response depends also on the viscoelastic properties of the adhered biomaterial, surface charges of adsorbed molecules and surface roughness. The modern QCM_D technology utilizing impedance measurement (as in the sensor introduced in references [3,12]) offers access to the resonance bandwidth in addition to resonance frequency. Bandwidth value is strictly connected with the viscoelastic properties of the sample [8,9]. Building upon the successful use of *in vitro* NAPPA- translated protein, Ramachandran *et al.* substituted the use of purified proteins with the use of cDNAs encoding the target proteins at each feature of the microarray [10]. The proteins were translated using a T7-coupled rabbit reticulocyte lysate *in vitro* transcription-translation (IVTT) system. Mammalian proteins can be expressed in a mammalian milieu, providing access to vast collections of cloned cDNAs. The addition of a *C*-terminal glutathione *S*-transferase (GST) tag to each protein enabled its capture on the array through an antibody to GST printed simultaneously with the expression plasmid [10,11]. In the present research, we coupled QCM_D and QCM_F with NAPPA technology [3,12], optimizing the monitoring in real time of the kinetics of the reaction to obtain information not only concerning the mass, but also on the viscosity of the sample and sensing the interaction among a query protein and the expressed protein.

The proteins monitored by the NAPPA-based nanogravimetric biosensor, namely p53 and MDM2, are of fundamental importance in the molecular mechanisms leading to malignant cell transformation and cancer; p53 is in fact a 53-kiloDalton phosphoprotein oncosuppressor, encoded by a 20-kilobases gene situated on the short arm of human chromosome 17 and termed as the “guardian of the genome” and the “policeman of oncogenes” [13,14]. Mutated, it is involved in up to 70% of human tumors, being responsible of cell growth arrest, senescence, apoptosis in response to an array of stimuli such as DNA damages (DSB, or double-strand-breaks), hypoxia, telomeres shortening, cell adhesion, oncogene activation and other molecular and cellular stresses [15]. MDM2, a p53-specific E3 ubiquitin ligase, inhibits p53 functions by binding to its *N*-terminal transcription-promoting domain, thus having an oncogenic activity. The MDM2-p53 plays a major role in cancer, being also a molecular therapeutic target and its monitoring is of crucial importance in cancer diagnosis and treatment [16].

In this report, our goal was finally to build the prototype of the enzyme-based biosensor for medical purposes, in which the immobilization procedure was carried out via Langmuir-Blodgett films. The enzyme implemented in our device [5] was laccase [6], which is a blue oxidase capable of oxidizing phenols and aromatic amines by reducing molecular oxygen to water by means of a full complement of copper atoms. Laccase belong to a large group of multicopper enzymes, which includes among others ascorbic acid oxidase and ceruloplasmine. They catalyze the oxidation of diverse compounds such as *o*-, *p*-diphenols, aminophenols, polyphenols, polyamines, lignin, some inorganic ions, aryldiamines, benzenthiols, and phenothiazines.

## 2. Materials and Methods

Taking into account all the considerations so far discussed and the three sensors recently introduced in the literature [2,3,5], in this section we summarize the techniques and procedures utilized in the construction of the three distinct prototypes.

### 2.1. Inorganic Sensor Based on Matrices of Calcium Oxide

The alternative long-term sampling method for the determination of environmental CO_2_ accumulation takes advantages of the properties of CaO to be carbonated by this gas. We carried out preliminary tests to assess the best concentration of CaO in the composite, individuated in the CaO/PEG weight ratio of 1/4, by studying the variation of mass connected to the carbonation process, in order to assess the quantity of gas absorbed in relation to the concentration of environmental CO_2_ by a fixed amount of composite. We tested the sensing properties of the composite materials via a nanogravimetric method by using a home-made glass chamber of 340 mL in volume [2]. The home-made chamber was provided with four input sockets able to arrange up to four quartzes at the same time, besides inlet and outlet valves to feed and empty the gas. As transducers, AT-cut quartz crystals were used, with a native frequency equal to 9.5 MHz, a blank diameter equal to 0.550″, an etched surface, an electrode diameter equal to 0.295″, with 100 Å Cr and 1000 Å Au as electrode materials (International Crystal Manufacturing Co, Inc., ICM, Oklahoma City, OK, USA). The preliminary experimental data highlighted that the composite was able to selectively detect CO_2_ via a nanogravimetric method by performing the experiments inside an atmosphere-controlled chamber filled with CO_2_[2]. Furthermore, the composite material showed a linear absorption of CO_2_ as a function of the gas concentration inside the atmosphere-controlled chamber, thus paving the way for the possible use of these matrices for applications in the field of sensor devices for long-term evaluation of accumulated environmental CO_2_. In reference [2] are illustrated the experimental results obtained from these experiments.

**Figure 1.**
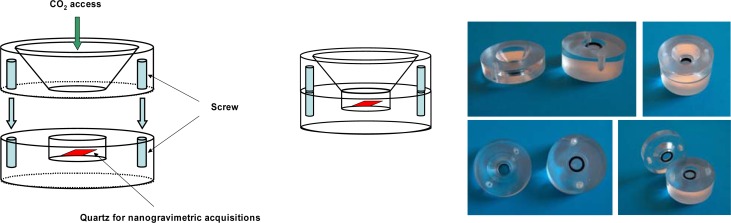
Illustration of the plastic cell containing the quartz for environmental CO_2_ long-term sampling, constructed in cooperation with Elbatech Srl, Marciana, Italy.

The previous reported considerations allowed us to design and realize a dosimeter for the long-term analysis of the carbon dioxide. We used the same transducers but they were inserted in a home-made and *ad ho*c designed and built Plexiglas measuring chamber, divided into two parts: the upper one, with a funnel opening, allowed the exchange of the sensing matrix with the environment and the lower one that allowed the housing of the transducer (Figure 1).

**Figure 2.**
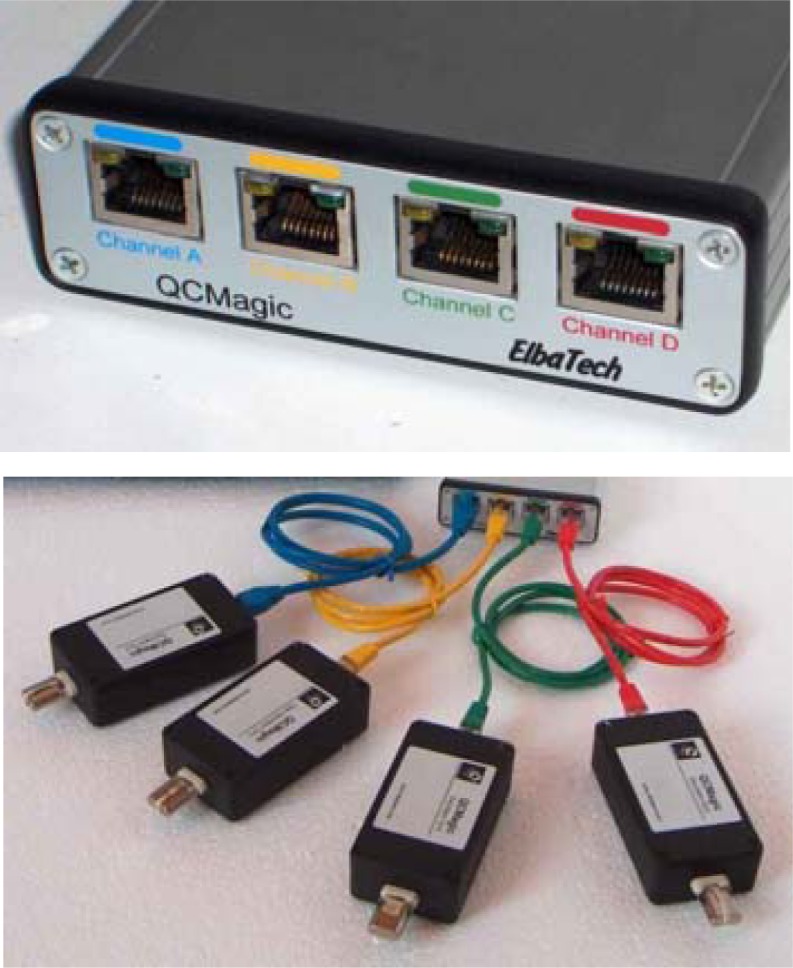
Nanogravimetric base-unit, able to drive up to four oscillating units (**Upper**). Oscillators unit connected via Ethernet cable to the base unit (**Lower**).

The nanogravimetric instrument used for relating CO_2_ concentrations with mass variations consisted of a base unit, interfaced to a PC via USB port and able to drive up to four oscillator units [2]. The base unit embedded the interface circuitry to/from the USB port, a digital signal controller and a fast programmable logic device containing an accurate four-channel counter, plus the four interfaces to the oscillator units. The counter logic was fully parallel, this meaning that the four input signals were acquired and counted-up simultaneously at a gate interval selectable from fractions of a second to 10 s. The base unit was powered by means of an external pluggable +12 V power supply which sustained input AC voltages from 90 to 240 VAC (Figure 2).

The choice to have the oscillators outside the base unit allows the maximum flexibility when building an experimental set-up. The noise immunity was preserved and guaranteed by the oscillator design, which was based on a precision internal reference crystal, used as a timebase comparator for the working quartz crystal. Only the mixed, lower frequency signal was then transmitted to the base unit by dedicated twisted pair lines on the cable. The oscillator units were connected to the base unit by means of standard Ethernet class V cables (see Figure 2 below).

The system was driven by an extremely user-friendly software running under MS-Windows™. It consisted of a lower level kernel, which implemented the necessary data transfer between the computer and the base unit, and of a higher level set of routines, which drove the user through an easy to perform data acquisition and analysis. In addition to the data acquisition service routine, a useful data display was implemented by which the user was easily able to retrieve the acquired signal values. Oscillating frequency *versus* time measurements can be performed following the data acquisition in real time on the computer screen, by means of strip chart plotting.

### 2.2. Biological Sensor Based on NAPPA and QCM_D Technology

**Figure 3.**
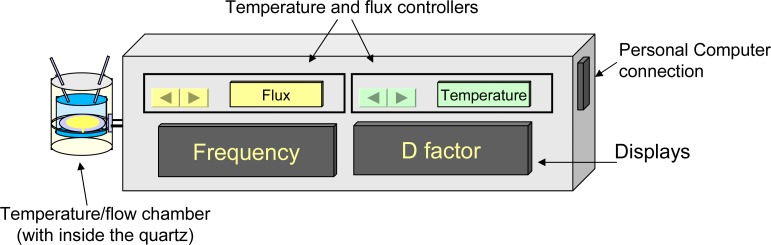
QCM_D biosensor prototype scheme. The quartz was positioned in a flow chamber that also guaranteed the temperature control. Temperature and flux rate were settable trough the controllers and D factor and frequency values are visible on two displays. On connecting the instrument to a PC it is possible to record the impedance curves, the frequency and D factor shifts in real time.

Our system, shown in Figure 3, detects the normalized dissipation factor of the quartz crystal by means of the “half-width half-height” of its impedance curve [3]. In our case, the quartz was connected to an RF gain-phase detector (Analog Devices, Inc., Norwood, MA, USA) and was driven by a precision Direct Digital Synthesizer (DDS, Analog Devices, Inc.) around its resonance frequency, thus acquiring conductance *vs.* frequency curve, which showed a typical Gaussian behavior [3].

**Figure 4.**
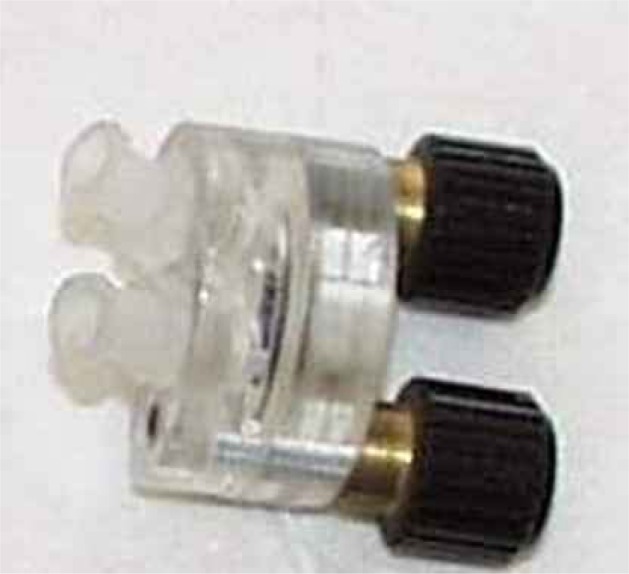
Plexiglas chamber utilized during the flow analysis of protein-protein interaction: the upper part, with the flow inlet and outlet, allowed the easy insertion of the biological fluid to be analysed and the lower allowed the proper housing of the quartz.

The curve peak was at the actual resonance frequency while the shape of the curve indicated how damped was the oscillation, *i.e.*, how the viscoelastic effects of the surrounding layers affected the oscillation. In order to have a stable control of the temperature, the experiments were conduced in a temperature chamber. The experimental set up, illustrated by the scheme in Figure 3, consisted of a temperature chamber were the quartz was positioned and monitored at the same time for frequency and dissipation factor variation. We designed a miniature flow-cell (Figure 4) to employ in protein-protein interaction analysis. The flow cell chamber volume was 100 μL and it was connected to a BioRad Econo Gradient Pump, able to pump solution in a flux range of 0.02–6 mL/min.

The transducer consisted of 9.5 MHz AT-cut quartz crystal of 14 mm blank diameter and 7.5 mm electrode diameter, produced by ICM. The electrode materials were 100 Å Cr and 1,000 Å Au. Microarrays were produced on the quartzes already described as highly sensitive transducers. Each quartz was printed with 4 × 4 NAPPA spots of 300 microns diameter, spaced 350 microns center-to-center [4], using the given genes and corresponding expressed proteins. The blank quartz was sent to the Harvard Institute of Proteomics (HIP) for the NAPPA spots printing as described in [3,4]; in order to express proteins we adopted the protocol described in [10]. Gene expression and protein synthesis took place at 30 °C for about 1.5 h; to prepare rabbit reticulocyte lysate mix (Expression Kit, Promega, Madison, WI, USA) we mixed 16 μL of TNT buffer, 8 μL of T7 polymerase, 4 μL of Cys, 8 μL of RNaseOUT (Invitrogen, Carlsbad, CA, USA), 160 μL of DEPC water (Ambion, Foster City, CA, USA), and 200 μL of reticulocyte lysate (TNT® T7 Coupled Reticulocyte Lysate System, Promega). Sixty μL of IVT lysate mix was added per quartz. The quartz, connected to the nanogravimeter inside the incubator, was incubated for 1.5 h at 30 °C for proteins synthesis and then, the temperature was decreased to 15 °C for a period of 0.5 hour to facilitate the proteins binding on the spot surface. The quartz was subsequently removed from the instrument and washed three times at room temperature (22 °C) in Milli-Q water. The quartz was then placed in the flux chamber for protein-protein interaction analysis. The protocol described above was followed identically for both control quartz (blank quartz) and working quartz. The proofs of principle to verify sensor response to NAPPA protein expression and immobilization were carried out immobilizing the following genes and corresponding expressed proteins: CDK2_Human gene, jun_Human gene and p53_Human gene [17]. As reference, a blank quartz was employed. For protein-protein interaction proof of principle, we tested the interaction between p53 proteins immobilized on the NAPPA surface (after its expression) with a MDM2 solution. In parallel experiments (submitted elsewhere as a result of a cooperation with the Biodesign Institute at the Arizona Institute of Proteomics) several other genes of significant clinical and biological implication and others configurations (10 × 10 spots) were tested. In particular CYP11A1_Human genes were immobilized and cholesterol was chosen for interaction analysis and their comparison with similar cholesterol sensors based on traditional technologies [18,19].

### 2.3. Biological Sensor Based on Langmuir-Blodgett Multi-Layers of Laccase

**Figure 5.**
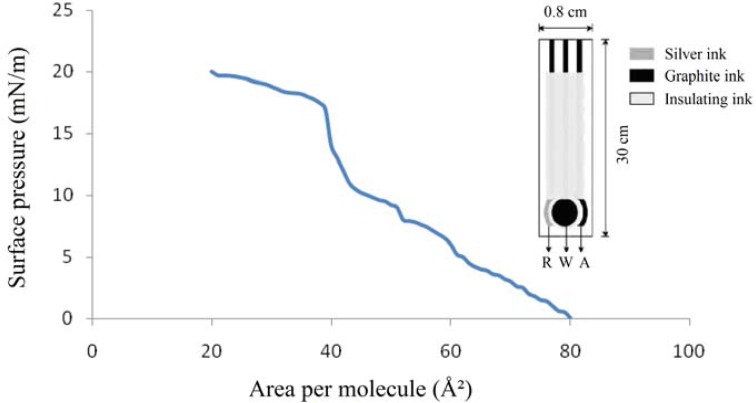
π-A isotherm of the laccase thin film at the air-water interface (**Left**). A couple of screen-printed electrodes used as a transducer based on graphite/ruthenium ink and the counter on silver one (**Right**).

A LB thin film [20] of recombinant laccase from *Rigidoporus lignosus* (formerly known as *Rigidoporus microporus*), obtained as previously reported [6], was prepared using a highly concentrated sample of laccase. The mixed chloroform solution in equimolar proportions had a concentration of 1 mg/mL. A volume of 50 μL of the mixture was spread on a Milli-Q water sub-phase (>17 MΩ) and the monolayer was compressed with movable barriers at a rate of 70 mm/minute. The deposition was of Y-type with a dipping rate of 25 mm/min. The drainage rate (in order to remove the film) was about 3.5 mm/min. The transfer pressure to obtain the LB film (Figure 5) was about 20 mN/m, at 22 °C.

The surface morphology and topology of the LB thin film of laccase was investigated via Atomic Force Microscopy (AFM) [5]. The roughness of the film was found to be 8.22 nm and the compressibility coefficient about 37.5 m/N as determined from the LB π-A isotherm at the air-water interface (Figure 5, Left). The enzyme was deposited onto the electrode via Langmuir-Schaefer (LS) technique [5] and a protocol of immobilization overnight was followed; after depositing the film, the electrodes (Figure 5, Right) were kept at 4 °C up to a maximum of 16 hours. The employed electrodes were ruthenium and graphite ones, while the counter-electrode was of silver. The area of the electrode was about 0.75 mm per 1 mm. The amperometric technique was used to polarize the electrochemical couple and to obtain a current discharge related to the amount of the investigated drug, namely clomipramine [5–21], with an EG & G PARC model 263A potentiometer, equipped with dedicated software.

## 3. Results and Discussion

### 3.1. Inorganic Sensor Based on Matrices of Calcium Oxide

**Figure 6.**
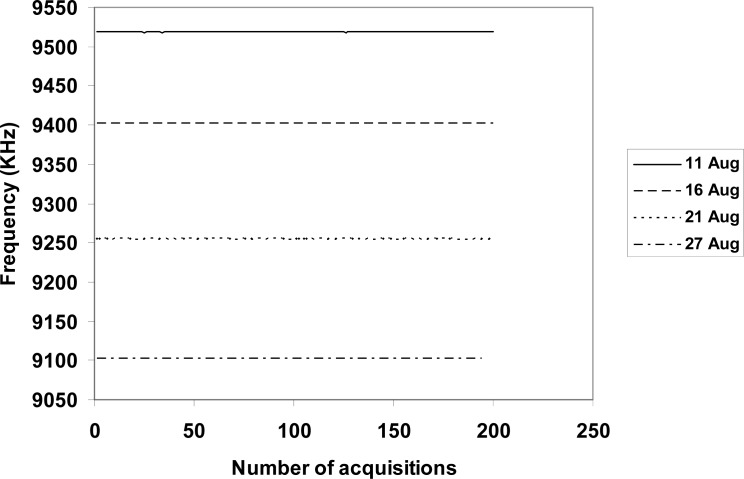
Variation of frequency in relation to the quantity of CO_2_ absorbed by the composite. Experimental data showed a linear absorption, coherently with the human activity of the sampling period.

We performed nanogravimetric acquisitions of frequency *vs.* time in order to assess the variation of mass at regular intervals of time after placing the small plastic cell in a room. We also measured the mass of the deposited sample at time zero, in order to have the value of frequency related to the composite before reacting with environmental CO_2_. We consequently carried out acquisitions of frequency *vs.* time in order to sample the indoor environmental CO_2_ in the room, illustrated in Figure 6.

Basing on our previous experiments [2], we were able to calculate the variation of mass *per* week and consequently the average quantity of CO_2_ in the environment. Experimental data, summarized in Table 1 for a clear vision, highlighted the variation of frequency in relation to the quantity of CO_2_ absorbed by the composite issued a linear absorption, coherently with the constant human activity of the sampling period. Specifically, we found the variation of mass in relation to the quantity of CO_2_ present in the environment (indoors), indicated the concentration of indoor CO_2_ during the sampling period was 10 times less than the average concentration in the atmosphere, thus indicating the good quality of the air in our laboratory.

Since the very good results were obtained from the usage of this long-term sampling device, we are presently programming new samplings both in indoor spaces affected by strong human activities and outdoors.

### 3.2. Biological Sensor Based on NAPPA and QCM_D Technology

**Figure 7.**
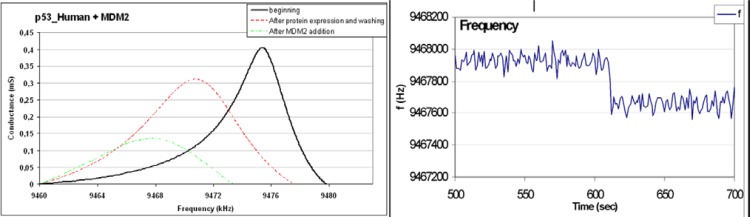
(**Left**) p53 quartz impedance curves recorded before the gene expression (solid curve), after the protein expression and the washing process (dashed curve) and after MDM2 addition (dotted curve); (**Right**) p53 quartz frequency variation in real time; at time t = 600 s a MDM2 solution (50 μM in PBS) was injected in the flow chamber.

The QCM_D results were calibrated both for frequency and D factor shifts using fructose [3,4]. We monitored by QCM-D the viscoelastic behaviour of the quartzes during the NAPPA expression process, recording the impedance curves in correspondence with the main steps of the expression process. Moreover we monitored by QCM-F the variation in the mass adsorbed on the surface of the sensors (corresponding to a decrease of the resonance frequency). Figure 7 Left shows the impedance curves of p53 quartz at different steps of the expression protocol, as following:

- At the beginning of the expression process, *i.e.*, before reticulocyte lysate addition;
- 120 min after reticulocyte lysate addition (*i.e.*, after 90 min at 30 °C for gene expression and protein synthesis plus 30 min at 15 °C for protein immobilization) and after washing process, at 22 °C;
- After the addition of a MDM2 50 μM solution.

In order to investigate the biosensor response to protein-protein interaction the p53 quartz, once expressed the proteins, was positioned in the flux chamber, connected with the pump, and a MDM2 solution was flowed on the quartz surface: 2 mL of 50 μM MDM2 solution in PBS was injected in the pump. The corresponding frequency decrease was recorded (Figure 7, Right).

In Table 2 are reported for p53 quartz impedance curves of Figure 7, Left, the values of the frequency and the half width at half height (Γ), along with the values of the variation in conductance Y_RE_ (mS). The ratio named normalized D factor, D_N_ = 2Γ/I, gives information on the shape of impedance curves and on sample viscosity, while the decrease in frequency *f* (Hz) is related to the amount of p53 molecules being immobilized.

The resulting Michaelis-Menten constants of the p53-MDM2 (calculated from the curve in Figure 7, Right) interaction appeared quite compatible with the literature.

The results mentioned before obtained using a human lysate and 10 × 10 spots per quartz reported in a separate communication (pending submission to a different journal) suggest that the NAPPA based biosensor functioned to monitor with high selectivity the single protein being expressed even in a mixture of different genes (as shown here in Figure 7, Right) and, from the analysis of D factor, allowed to acquire in real time information on the characteristics both of single protein being expressed with unique signature and on the kinetic constant of the reaction.

In order to properly set-up the QCM_D for routine measurements, we are going to immobilize the NAPPA on the given reference quartzes provided by the manufacturer [4]. Then, in order to eliminate the background signal, we are going to routinely carry out the measurements starting from the crystal native frequency subtracted of 15 kHz and using a step of 1.0 Hz for collecting whole impedance plots at the necessary resolution and with a considerable number of data points. For this reason, we have used a dsPIC (Microchip Technology, Inc., Chandler, AZ, USA) featuring at the same time good computational power and sufficient memory space. This represents a PC-driven prototype to establish the proof of principle. The industrial prototype is being designed and realized, under a different contract with significant larger amount of money than here provided by the MIUR under the indicated FIRB contract, in order to have a temperature-controlled *ad hoc* chamber, hardware and software optimized to increase speed, computational power and compactness to incorporate hardware, in order to produce a finalized friendly stand-alone device.

### 3.3. Biological Sensor Based on Langmuir-Blodgett Layers of Laccase

**Figure 8.**
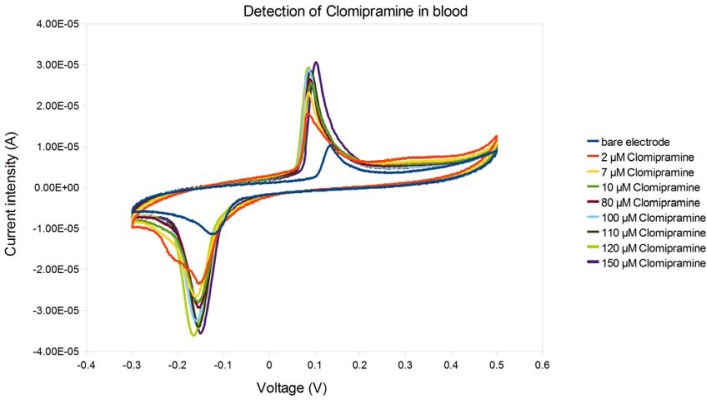
Cyclic voltammetry of laccase-based biosensor for detection of clomipramine at increasing concentrations in human blood.

Clomipramine [21,22], a drug belonging to the tricylic tertiary amine antidepressants class, is widely used for the therapy of depressive and obsessive disorders. Because of its clinical importance many analytical methods have been developed to monitor its levels, above all chromatographic techniques (gas chromatography, high performance liquid chromatography), eventually coupled with tandem mass spectrometry, but all these techniques are time-consuming and laborious. Moreover, the AGNP-TDM panel of experts has emphasized the importance of therapeutic drug monitoring [23]. In our experiment, clomipramine was added at varying concentrations in the micromolar range. The therapeutic dose is from 75 mg/day to 200 mg/day; the pharmacokinetics among patients is extremely variable. Generally, the therapeutic concentration in the human blood of psychiatric patients is usually in the low micromolar range. The side-effects of the drugs, especially in case of overdose, are seizures, hematological, cardiological and neurological adverse effects up to the coma (tricyclic antidepressant syndrome, [24]). Experiments carried out with cyclic voltammetry (see Figure 8) highlighted excellent reproducibility and linearity of the peaks of oxidation and reduction, related to the presence of the drug in several biological fluids (in particular in whole blood).

**Figure 9.**
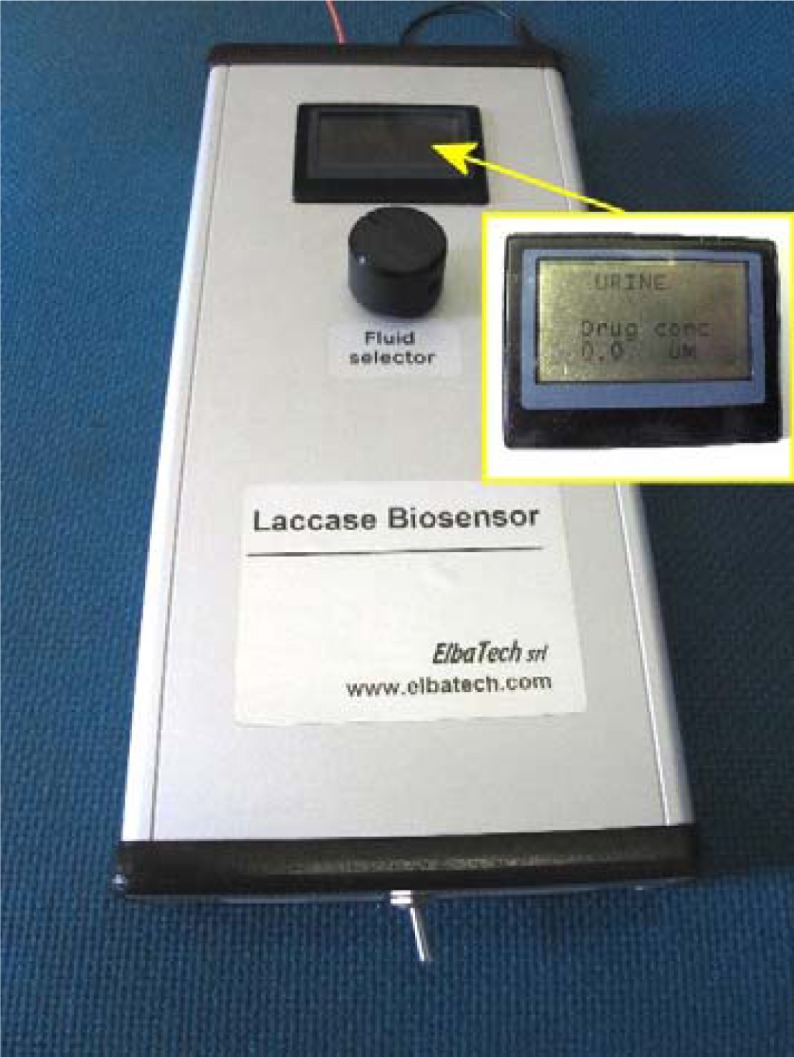
Prototype of laccase sensor jointly constructed with Elbatech Srl, Marciana (LI), Italy, compact and portable for all fluids indicated in Table 3.

These results allowed the design and the creation of a prototype of a biological sensor for antidepressants. The instrument consisted of a central unit, able to bias the working electrode on which the enzyme was deposited and to detect the current generated as a result of the interaction of the enzyme with the analyte containing the drug of interest. By a multiple selector, the user can choose the fluid to be analyzed and, through the proper calibration parameters, on the display, the proper drug concentration will be provided. The instrument was powered by two 9V batteries, in order to avoid noise from the mains voltage (see Figure 9).

Use of commercial laccase, screen-printed electrodes technique and a portable device appear to provide an emerging instrument suitable for investigation in biological fluids as well as for other medical applications. The same enzyme and the same proposed device could be used also in many different fields, such as in degradation of polyaromatic hydrocarbons, in textile industry, in food industry and in waste detoxification. Laccase-based sensors for detection of clomipramine in breast milk, saliva and semen were less sensitive than the others, while sensors for the monitoring of the drug level in urine and blood had better pronounced and separated peaks (as shown in Table 3).

Moreover, if we compare these results with our previous findings we can study how changes in thickness and in number of layers can increase the functionality of a biosensor: the sensitivity of the biosensor in blood with LB 3-layers was more than the double of the value found here with only one layer. It is known in fact that highly ordered structured biofilm can increase the sensitivity and the electron kinetics transfer (difference in the width between the oxidation and reduction peaks).

## 4. Conclusions

In summary, our resulting prototypes appear to yield satisfactory proof of principles in the shown specific health and environmental applications. Indeed, to measure CO_2_, we have realized a new device based on the use of a sensor technology (such as the nanogravimetric one) and an array of capture, highly specific for the gas of interest. Calcium oxide may be considered a valid solution to solve the problem of the detection of carbon dioxide due to its ability to selectively absorb this gas through a chemical carbonation reaction. The carbonation reaction leads to a substantial variation of the molecular weight and was therefore taken into account in the manufacture of gravimetric detection devices. The characteristics of the reaction allowed the construction of a dosimeter for the long-term analysis of the carbon dioxide, competitive with respect to the devices already available on the market based on infrared measurements (CO_2_ reduces the incidence of infrared radiation on the sensor, then, depending on the concentration of CO_2_) or on measurements of the variations of a voltage across a solid electrolyte depending on the concentration of CO_2_.

For what concerns the NAPPA QCM_D conductance device the results presented demonstrated a valid response for the protein-protein interaction analysis, exploiting the great advantage of this technique that allowed the real-time, label-free characterization of molecular binding kinetics to an immobilized receptor. A proof of principle was realized immobilizing p53 plasmid, resulting in a biosensor for MDM2. The most challenging prospective of the innovative biosensors emerging from this technology is the potential capability to develop a large number of sensors for molecules of biological and medical interest, by simply changing the cDNA immobilized on the sensor, without changing the detection technology. Among the avenues being presently explored NAPPA-based vaccines identification appears to represent an additional promising future perspective in the frame of the new OMICS-based Public Health. Vaccinology has emerged as a complex interdisciplinary science, especially because of the contributions of the new OMICS disciplines [25]. In addition to what was anticipated some time ago [26], only recently were protein arrays used to discover new antigenic determinants for vaccine development [27,28]. NAPPA-based sensors could be used for screening the affinity between the identified proteins and the immunological synapse (CD4, TCR, MHC complex). Affinity kinetics can be evaluated also using classical techniques, or new efforts to evaluate it via Atomic Force Microscopy (AFM) and Surface Plasmon Resonance (SPR). In the right column of Table 4 are shown the genes that interact with immunological human synapse (CD4 + TCR + MHC).

Finally, with the designed and realized amperometric sensor [5], in order to offer a suitable instrument for routine medical application, we used only one layer of commercial laccase to fully characterize clomipramine pharmacokinetics in different biological fluids of relevant medical interest, namely human blood, saliva, urine, breast-milk, semen and cerebro-spinal fluid or liquor (CSF).

As far as we know, this is the first comprehensive characterization of clomipramine concentration in different biological fluids of medical interest, since drug monitoring in different biological samples is very important. Biological fluids were taken from healthy donor volunteers, who gave their informed consent, and analyzed immediately after being collected. The motivation of studying electrochemical behavior of clomipramine in different biological fluid of clinical interest was to provide a comprehensive pharmacokinetic profile. Schimmell *et al*. [29,30] studied drug plasma levels in a breast-fed infant whose mother had taken clomipramine during pregnancy and continued after giving birth. They found that levels were high following delivery but decreased gradually and were at the lowest detectable concentration at 35 days, even though breast-feeding continued. Clomipramine has been used by breast-feeding mothers without adverse effects on the newborn, even if drug monitoring in human breast milk is of crucial importance. Urine is a biological flood that can be easily obtained with great acceptability from the patient and can help in rapid assessment and screening in emergency situations [31]. CSF fluid can be exploited for monitoring the neurochemical changes during therapy or disease [32,33]. As far as semen is concerned, there were some communications of a relationship between clomipramine level in sperm and male infertility: clomipramine seems to modify sperm motility in a significant way, but these findings are controversial and need to be confirmed by further research. Moreover, clomipramine concentrations in blood, urine and liquor correlate with patient response state, which is the most important clinical parameter for the assessment of drug functionality and working.

## Figures and Tables

**Table 1.** Variation of mass in relation to the quantity of CO_2_ present in the environment (indoor). The concentration of indoor CO_2_ was found 10 times less than the average concentration in the atmosphere, thus indicating good quality of the air.

**Sampling Period (days)**	**Variation of Mass Δm (μg)**	**Concentration of Environmental CO_2_ (ppm)**
7	0.57 ± 0.007	48 ± 1
7	0.72 ± 0.007	55 ± 1
7	0.75 ± 0.007	55 ± 1

**Table 2.** Peak frequency (*f*), half-width at half-height (*Γ*), maximum conductance increment (*Y_RE_*), and normalized *D* factor for the impedance curves shown in Figure 7 Left.

**Impedance Curve**	***f* (Hz)**	***Γ (Hz)***	***Y_RE_ (mS)***	***D_N_ (Hz/mS)***
Beginning	9475435	2220	0,415	10699
After protein expression and washing	9470905	3705	0.310	23903
After MDM2 addition	9467860	3600	0.135	53333

**Table 3.** Peak potentials and sensitivity of Laccase-based biosensor measured at increasing concentrations in different human biological fluids.

**Fluid**	**Peak (mV)**	**Sensitivity**
Blood	+75; −150	191.91 mA/M
Urine	+100; −150	138.93 mA/M
Liquor	+100; −200	31.70 mA/M
Breast-milk	+100; −200	9.80 mA/M
Saliva	+150; −100	11.14 mA/M
Sperm	+110; −220	19.00 mA/M

**Table 4.** Genes which interact with immunological human synapses.

**Microorganism**	**Antigen**
Pseudomonas aeruginosa	PA0044, PA0807, PA0973 (OprL), PA1080, PA1148, PA1248, PA2300, PA3407, PA3724, PA3841 (ExoS), PA3931, PA4110, PA4922, PA5369, FlicA, OprI, OprH2, OprE, OprF, exotoxin A, flagellin
Vibrio cholerae	Protein 1 (VC1085), 2 (VC2283), 3 (VC1893), 4 (VC2261), 5 (VC0339, PSD), 6 (VC1494), 7 (VC0556), 8 (VC0975)
Yersinia pestis	VA (V antigen), F1 antigen
Francisella tularensis	O-antigen
Bacillus anthracis	PA (protective antigen)
